# Beta Blocker Toxicity at Therapeutic Doses: A Look Into Carvedilol Use in the Elderly

**DOI:** 10.1002/ccr3.72891

**Published:** 2026-06-06

**Authors:** Samuel Amo‐Tachie

**Affiliations:** ^1^ University of Ghana Medical School University of Ghana Accra Ghana

**Keywords:** beta‐blocker toxicity, carvedilol, elderly patient, therapeutic‐dose toxicity

## Abstract

Beta‐blocker toxicity does not occur only in overdose situations. In vulnerable populations such as children and the elderly, therapeutic doses may produce cumulative toxic effects similar to those seen in acute overdose. These patients require monitoring that extends beyond basic laboratory investigations to preempt cumulative toxicity.

## 1 Introduction

Beta blockers, specifically bisoprolol, metoprolol, and carvedilol, are among the drugs, alongside renin‐angiotensin‐aldosterone system (RAAS) inhibitors, mineralocorticoid receptor antagonists, and sodium‐glucose cotransporter‐2 (SGLT2) inhibitors, that are considered mortality‐improving therapies in heart failure. In compensated heart failure, these beta blockers are useful across all stages [1, 2]. Carvedilol is commonly used because of its additional alpha‐1 receptor‐blocking activity, which further decreases blood pressure through both preload and afterload reduction.

Toxicity is most commonly caused by overdose but may also occur at therapeutic doses, although rarely, particularly in situations such as liver failure. It is known in children that a single therapeutic adult dose of a beta blocker may be sufficient to induce toxicity [3, 4]. Since the elderly and children often share similar risk factors due to declining organ function and immature organ systems, respectively, such cases may also occur in elderly patients. This article discusses a case of beta‐blocker toxicity in a 90‐year‐old man following therapeutic use of carvedilol.

## 2 Case History and Examination

This is the case of a 90‐year‐old man, a non‐smoker and non‐alcoholic, known to have heart failure secondary to hypertensive heart disease. He was compliant with his medications, which included lisinopril 10 mg daily, spironolactone 25 mg daily, furosemide 40 mg daily, and carvedilol 6.25 mg daily. He had been taking these medications for approximately 6 months.

He presented to the emergency room on two separate occasions within 1 week for recurrent hypoglycemic episodes, which were initially attributed to poor feeding habits, as he preferred occasional snacks to proper meals, for which he had little appetite. His first hypoglycemic episode (3.1 mmol/L) was accompanied by a seizure. This was corrected with dextrose infusions, and his basic metabolic panel was unremarkable.

On his second admission, he again presented with severe hypoglycemia (2.7 mmol/L), this time associated with altered mental status and respiratory depression. On examination, his Glasgow Coma Scale (GCS) score was 10/15, and his pupils were equally reactive to light. His respiratory rate was 12 cycles per minute, with reduced air entry on auscultation but no crepitations, and oxygen saturation was 92% on room air. He was bradycardic and hypotensive, with a pulse rate of 56 beats per minute and blood pressure of 77/59 mmHg. His pulse was faint but regular, and heart sounds were normal.

However, he was neither jaundiced nor febrile, had satisfactory hydration, and mild bilateral pedal edema. Examination of his prescription pills showed no evidence of excessive intake, as every pill had been taken according to schedule. This was also confirmed by his caretakers, who ensured timely and correct administration of his medications.

## 3 Differential Diagnosis

Based on these findings, the differential diagnoses included severe acute pneumonia, acute decompensated heart failure, anorexia‐related hypoglycemia, and beta‐blocker toxicity. In the absence of fever, cough, suggestive chest findings, and later radiological evidence supporting these differentials, the diagnosis of beta‐blocker toxicity was favored.

## 4 Outcome and Follow‐Up

The patient was supported with oxygen therapy and resuscitated with dextrose and saline infusions, which achieved normoglycemia and normotension; however, he still had altered mental status with only mild improvement in GCS to 12/15.

He was also started on intravenous amoxiclav followed by azithromycin after ECG review excluded QT prolongation, as a pneumonia was yet to be fully ruled out, and aspiration was a concern due to his reduced GCS score. Monitoring during admission revealed recurrent episodes of hypoglycemia, bradycardia, and hypotension.

A chest X‐ray subsequently done showed cardiomegaly with clear lung fields, while ECG findings suggested left ventricular hypertrophy following resuscitation. Renal function tests showed elevated urea and creatinine levels (13.0 and 132 mmol/L respectively) with estimated glomerular filtration rate (eGFR) of 40.6 mL/min/1.73 m^2^, while electrolytes remained normal. Complete blood count revealed only mild anemia with hemoglobin of 10.1 g/dL. Liver function tests were unremarkable. With these findings, beta‐blocker toxicity became the leading unifying diagnosis.

Using the Naranjo Scale for adverse drug reactions (Table 1) [5], the patient scored 8/13, suggesting carvedilol as a probable cause, since additional criteria such as serum drug levels, re‐challenge, and placebo administration were not applicable. Similarly, application of the World Health Organization–Uppsala Monitoring Centre (WHO‐UMC) causality assessment system classified carvedilol as a probable/likely cause of the adverse reaction (Table 2) [6].

**TABLE 1:** Naranjo adverse drug reaction probability scale.

#	Question	Answer	Score
Q1	Are there previous conclusive reports on this reaction?	Yes	+1
Q2	Did the adverse event appear after the suspected drug was administered?	Yes	+2
Q3	Did the adverse reaction improve when the drug was discontinued?	Yes	+1
Q4	Did the adverse reaction reappear upon re‐administration?	Not done	0
Q5	Are there alternative causes that could have caused the reaction?	No	+2
Q6	Did the reaction reappear when a placebo was given?	Not done	0
Q7	Was the drug detected in toxic concentrations in blood?	Do not know	0
Q8	Was the reaction worsened by increased dose or lessened by decreased dose?	Yes	+1
Q9	Did the patient have a similar reaction to the same or similar drugs?	No	0
Q10	Was the adverse event confirmed by objective evidence?	Yes	+1
Total			8

**TABLE 2:** WHO‐UMC causality categories.

Category	Criteria
Certain	Event or laboratory test abnormality, with plausible time relationship to drug intake Cannot be explained by disease or other drugs Response to withdrawal (dechallenge) plausible Event definitive pharmacologically or phenomenologically Rechallenge satisfactory (if necessary)
Probable/Likely	Event with reasonable time relationship to drug intake Unlikely to be attributed to disease or other drugs Clinically reasonable response to withdrawal (dechallenge) Rechallenge not required
Possible	Event with reasonable time relationship to drug intake Could also be explained by disease or other drugs Information on drug withdrawal may be lacking or unclear
Unlikely	Event with a time relationship to drug intake that makes a causal relationship improbable Other drugs, chemicals, or underlying disease provide plausible explanations
Conditional/Unclassified	Event reported but more data needed for proper assessment Additional information under examination
Unassessable/Unclassifiable	Report suggests an adverse reaction Cannot be judged because information is insufficient or contradictory Data cannot be supplemented or verified

Carvedilol and the patient's other medications were withheld, and intravenous glucagon 3 mg hourly was initiated. There was a rapid response to IV glucagon; by the second dose, the patient had regained full consciousness with GCS 15/15, and within 12 h he was successfully weaned off oxygen.

Within 24 h, his average blood pressure stabilized around 135/85 mmHg, allowing lisinopril and spironolactone to be resumed. He was discharged on the third day of admission after effective monitoring. Repeat renal function testing showed improvement: urea 11.4 mmol/L, creatinine 110 mmol/L, and eGFR 50.6 mL/min/1.73 m^2^.

## 5 Discussion

Carvedilol is a non‐selective beta‐adrenergic blocker (β1 and β2) with additional alpha‐1 adrenergic blocking activity, making it distinct from many other beta blockers. This dual mechanism underlies its hemodynamic benefits, especially in patients with heart failure and hypertension.

Beta‐1 blockade reduces heart rate (negative chronotropy) and myocardial contractility (negative inotropy), thereby lowering cardiac output and myocardial oxygen demand. There is also an antiarrhythmic effect through reduced atrioventricular nodal conduction. Additionally, beta‐1 blockade inhibits renin release from juxtaglomerular cells, suppressing activation of the renin‐angiotensin‐aldosterone system (RAAS) [7].

Beta‐2 blockade may cause bronchospasm and vasoconstriction, although this is partially offset by the alpha‐1 blockade of carvedilol, which produces vasodilation and consequently reduces systemic vascular resistance and venous return, decreasing both preload and afterload [8].

Reduced hepatic glycogenolysis, leading to hypoglycemia, and decreased skeletal muscle sensitivity to insulin also result from β2 blockade. Carvedilol additionally exhibits antioxidant properties and inhibits vascular smooth muscle proliferation, thereby providing further cardiovascular protection [9].

The drug is extensively metabolized by hepatic cytochrome P450 enzymes, particularly CYP2D6 and CYP2C9, and is more than 98% protein bound, mainly to albumin [10].

Clinical manifestations of beta‐blocker toxicity include bradycardia, hypotension, central nervous system depression, delirium, respiratory depression, seizures, and bronchospasm [11]. As seen in this case, carvedilol toxicity, although uncommon at therapeutic doses, may still occur in vulnerable populations such as the elderly. This may occur through a myriad of factors, including age‐related pharmacokinetic changes, drug accumulation, pharmacogenetic variation, autonomic dysfunction, masking of hypoglycemia, and similarities to pediatric physiology.

The patient's presentation with bradycardia, hypotension, central nervous system depression, respiratory depression, and seizures strongly supported beta‐blocker toxicity as the primary diagnosis. Although lisinopril may cause hypoglycemia in diabetic patients on insulin or insulin secretagogues through enhanced insulin sensitivity, this patient was neither diabetic nor receiving such medications [12].

Despite normal liver function tests, reduced hepatic metabolic capacity could not be excluded. Aging is associated with reduced hepatic blood flow and diminished enzyme activity, changes that may not be reflected in routine liver function tests [13].

Furthermore, elderly or malnourished individuals with reduced serum albumin levels may have increased proportions of unbound carvedilol, thereby potentiating its pharmacological and toxic effects [14].

Genetic polymorphisms affecting CYP2D6, although less common in African populations, may impair carvedilol metabolism [15, 16]. Elderly individuals are also more likely to exhibit poor metabolizer phenotypes, predisposing them to drug accumulation despite standard dosing [17]. This patient had been taking carvedilol for approximately 6 months, which may have allowed sufficient time for cumulative toxicity to develop.

Reduced baroreceptor sensitivity and impaired autonomic responsiveness associated with aging further diminish compensatory responses to beta‐blocker‐induced bradycardia and hypotension [18].

Beta blockers also inhibit sympathetic warning signs of hypoglycemia such as tachycardia and tremors, thereby delaying recognition and treatment. Carvedilol additionally impairs gluconeogenesis and glycogenolysis despite its mixed receptor activity [19].

The similarities in susceptibility between elderly and pediatric populations likely arise from declining organ function in the elderly and immature organ systems in children. Toxicity at therapeutic doses has been documented in pediatric patients, and similar mechanisms may explain this presentation [4].

The patient's rapid response to intravenous glucagon further supported beta‐blocker toxicity as the primary etiology. Glucagon increases intracellular cyclic adenosine monophosphate (cAMP) through non‐adrenergic pathways, thereby improving cardiac activity. It also increases blood glucose through enhanced gluconeogenesis and glycogenolysis [20].

Due to these counteractive mechanisms that bypass autonomic pathways, glucagon serves as an effective antidote in beta‐blocker toxicity, often more effectively than atropine.

Carvedilol may also influence renal function and central nervous system activity, especially in elderly individuals. In predisposed patients with hypotension, heart failure, or reduced renal perfusion, carvedilol may contribute to worsening renal function through its antihypertensive and negative inotropic effects, which reduce renal perfusion pressure and glomerular filtration rate [21].

Being highly lipophilic, carvedilol readily crosses the blood–brain barrier more than hydrophilic beta blockers. This increases the risk of central nervous system adverse effects such as fatigue, dizziness, depression, hallucinations, and confusion, especially in elderly individuals [22].

Carvedilol also possesses membrane‐stabilizing activity similar to propranolol, one of the most toxic beta blockers, while also being slightly more lipid soluble [23]. Membrane‐stabilizing activity functions similarly to sodium‐channel blockade seen with Class IA antiarrhythmic agents and increases the propensity for seizure activity.

This patient experienced seizures at a relatively moderate level of hypoglycemia (3.1 mmol/L), a level that does not typically induce neuroglycopenic seizures in non‐diabetic patients [24, 25, 26]. He had also experienced prolonged anorexia and fatigue, symptoms that may be better explained by central nervous system toxicity.

Lipophilicity facilitates accumulation of the drug within the CNS, leading to neurological manifestations that may be accentuated by age‐related blood–brain barrier dysfunction. The combined effects of lipophilicity and membrane‐stabilizing activity likely potentiated the patient's CNS symptoms.

## 6 Conclusion

This case highlights the need for careful consideration of carvedilol dosing in elderly patients. Despite normal liver function tests, age‐related metabolic changes, variability in protein binding, and genetic differences may predispose elderly individuals to significant toxicity even at standard therapeutic doses.

Clinicians should maintain a high index of suspicion for adverse drug reactions in this population and consider functional assessments beyond routine laboratory investigations, particularly in vulnerable groups such as the elderly. Prompt recognition and treatment of beta‐blocker toxicity are critical in reducing morbidity and mortality.

## Author Contributions

**Samuel Amo‐Tachie:** conceptualization, data curation, formal analysis, investigation, methodology, resources, supervision, writing – original draft, writing – review and editing.

## Funding

The author has nothing to report.

## Consent

A written informed consent was obtained from the patient's representative to publish this report in accordance with the journal's consent policy.

## Conflicts of Interest

The author declares no conflicts of interest.

## Data Availability

The data that support the findings of this study are available on request from the corresponding author (S.A.‐T.).
